# Clinical features and prognostic factors of critically ill patients with COVID-19 in Daegu, South Korea: A multi-center retrospective study

**DOI:** 10.1097/MD.0000000000024437

**Published:** 2021-02-19

**Authors:** Eun Jin Kim, Yong Hoon Lee, Jae Seok Park, Jaehee Lee, Shin Yup Lee, Yeonjae Kim, Yong Shik Kwon, Jong Geol Jang, Kyeong-Cheol Shin, Kyung Chan Kim, Eun Young Choi

**Affiliations:** a Division of Pulmonary and Critical Care Medicine, Daegu Catholic university hospital; b Department of Internal Medicine, School of Medicine, Kyungpook National University; c Division of Pulmonology, Department of Internal Medicine, Keimyung University Dongsan Hospital, Keimyung University School of Medicine; d Division of Pulmonary and Critical Care Medicine, Kyungpook National University Chilgok Hospital; e Division of Pulmonary and Critical Care Medicine, Deagu Fatima Hospital; f Division of Pulmonary and Critical Care Medicine, Yeungnam University Medical Center, Daegu, Republic of Korea.

**Keywords:** COVID-19, critically ill

## Abstract

To describe the clinical and demographic characteristics of critically ill patients with COVID-19 in Daegu, South Korea, and to explore the risk factors for in-hospital mortality in these patients.

Retrospective cohort study of 110 critically ill patients with COVID-19 admitted to the ICU in Daegu, South Korea, between February 18 and April 5, 2020. The final date of follow-up was April 20, 2020.

A total of 110 patient medical records were reviewed. The median age was 71 years (interquartile range [IQR] = 63–78 years). During the study period, 47 patients (42.7%) died in the hospital. The most common SARS-CoV-2 infection related complication was acute respiratory distress syndrome (ARDS) in 95 patients (86.4%). Of the 79 patients (71.8%) who received invasive mechanical ventilation, 46 (58.2%) received neuromuscular blockade injection, and 19 (24.1%) received ECMO treatment. All patients received antibiotic injection, 99 patients (90%) received hydroxychloroquine, 96 patients (87.3%) received lopinavir-ritonavir antiviral medication, and 14 patients (12.7%) received other antiviral agents, including darunavir-cobicistat and emtricitabine-tenofovir. In the multivariable logistic regression model, the odds ratio of in-hospital death was higher with APACHE II score (OR = 1.126; 95% CI = 1.014–1.252; *P* *=* .027).

The in-hospital mortality rate of critically ill patients with COVID-19 was approximately 40%. Higher APACHE II score at admission was an independent risk factor for death in these patients.

## 1 Introduction

On March 11, 2020, the World Health Organization (WHO) declared the coronavirus disease 2019 (COVID-19) a pandemic. In South Korea, the first confirmed case of COVID-19 was reported on January 20, 2020.^[1]^ In the city of Daegu, the fourth largest city of South Korea with 2.5 million residents, the first COVID-19 case was confirmed on February 18, 2020. Within days, the number of cases surged due to the community transmission within a secretive religious organization. Health care facilities and government officials in Daegu rapidly responded to the outbreak and conducted widespread testing for severe acute respiratory syndrome coronavirus 2 (SARS-CoV-2) in suspected patients. As of April 20, 2020, the majority of COVID-19 cases in South Korea were identified in Daegu (64.02%, 6833 patients).^[2]^

Recent reports from China, Italy and Unites States suggest that the characteristics and mortality rate of critically ill patients with COVID-19 vary among countries.^[3–5]^ However, there have been no reports on the characteristics and mortality rate of critically ill patients with COVID-19 in a country with widespread virus-testing, such as South Korea.

The objective of this multi-center retrospective study was to describe the clinical characteristics, and to investigate the risk factors for in-hospital mortality, of critically ill patients with COVID-19 in Daegu, South Korea.

## 2 Methods

### 2.1 Study population and Setting

We retrospectively reviewed the medical records of all adult patients with laboratory-confirmed SARS-CoV-2 infection who were subsequently admitted to one of the intensive care units (ICUs) at the 7 tertiary or referral hospitals in Daegu, South Korea, between February 18 and April 5, 2020. According to the WHO guidance, laboratory confirmation for SARS-CoV-2 was defined as a positive result on real-time reverse transcription-polymerase chain reaction (RT-PCR) assay of nasal and pharyngeal swabs. During the study period, all adult patients (age ≥18 years) with confirmed SARS-CoV-2 infections admitted to the ICUs via the emergency or outpatient department were eligible for inclusion. This study was conducted in accordance with the tenets of the Declaration of Helsinki and was reviewed and approved by the institutional review board of Yeungnam University Hospital (YUH IRB 2020–03–057). The requirement for informed consent was waived due to the retrospective study design.

### 2.2 Data collection and definitions

Data were reviewed from electronic medical records using a structured form that was completed by the treating physician. The form collected demographic data, comorbid conditions, and clinical characteristics, antiviral treatment data, mode of respiratory support (invasive mechanical ventilation, non-invasive mechanical ventilation, high-flow nasal cannula, oxygen mask), level of positive end-expiratory pressure (PEEP), fraction of inspired oxygen (FIO2), arterial partial pressure of oxygen (PaO2), PaO_2_/FIO_2_ ratio, the use of extracorporeal membrane oxygenation (ECMO), prone positioning, and clinical outcome (in-hospital overall mortality). Illness severity was calculated using the Acute Physiological and Chronic Health Evaluation (APACHE) II, Sequential Organ Failure Assessment (SOFA) scores, CURB-65 severity scores and National Early Warning Scores (NEWS). Acute respiratory distress syndrome (ARDS) was defined according to the Berlin definition.^[6]^ Additionally, in order to identify early stage ARDS for which mechanical ventilation is not required, patients who did not require a PEEP of 5 cm H_2_O or more were considered to have ARDS if they otherwise met the Berlin definition of ARDS. Septic shock was defined according to the third international consensus definitions for sepsis and septic shock (Sepsis-3).^[7]^ Acute cardiac injury was diagnosed if serum concentrations of cardiac troponin I or T were above the upper limit of the reference range (>0.04 npg/ml). Acute kidney injury (AKI) was identified using the definition of the Acute Kidney Injury Network ^[8]^: an increase in the serum creatinine level to ≥0.3 mg/dl, an increase in baseline serum creatinine level to ≥150%, or the initiation of dialysis without a history of chronic kidney disease. The number of patients who had died, been discharged, and were still admitted in the hospital as of April 20, 2020, were recorded.

### 2.3 Statistical analysis

Descriptive analyses of the variables were expressed as medians (interquartile range, IQR) or totals (%). Patient characteristics were compared between outcome groups and were reported as the differences with 95% confidence intervals (CIs). Categorical data were compared using Chi-Squared or Fisher exact tests. Non-normally distributed continuous data were compared using Mann–Whitney–Wilcoxon tests. Bivariate logistic regression analysis for in-hospital death was performed to investigate the risk factors associated with in-hospital death. Variables for the multivariate analysis were selected based on the significance of the differences between the 2 groups. Variables with less clinical significance, missing values or interrelated variables were excluded. Variables applicable only to certain subgroups of patients were also excluded (e.g., neuromuscular blockade for use in patients receiving mechanical ventilation only). Considering the subjective nature of the diagnostic criteria and the possibility of ARDS under-diagnosis,^[9]^ especially in patients who do not receive invasive mechanical ventilation, ARDS was excluded. Instead, the PF ratio was converted into a categorical variable with a cut-off value of 150 and included in the multivariate analysis. The variables selected for multivariate analysis included diarrhea, APACHE II score, blood urea nitrogen (>17.6 mg/dl), glucose (>176 mg/dl), PF ratio (≤150), as well as the use of other antiviral agents. The cut-off values for laboratory tests were determined using the receiver operating characteristic curve analysis. In all analyses, *P* values <.05 were considered statistically significant when two-tailed tests were performed. All statistical procedures were performed using SPSS software (version 24.0, SPSS Inc., Chicago, IL).

## 3 Results

Daegu city in South Korea is a SARS-CoV-2 outbreak area, with 6833 confirmed cases within the 65 days between the first confirmed patient on February 20, 2020, and the end of our study registration on April 5. A total of 110 patients were admitted to the ICU at one of the 7 tertiary or referral hospitals in Daegu. The median duration of follow-up was 28.5 days (IQR = 15.8–42.0 days).

### 3.1 Baseline characteristics

The median age of 110 patients was 71 years (IQR = 63–78 years). There were 67 male (70%) patients and 5 patients (5%) were current smokers. In total, 47 patients (42.7%) died in the hospital during the study period. The most common comorbidity was hypertension observed in 55 patients (50%), followed by diabetes, chronic kidney disease, dementia, cerebrovascular disease, malignancy, cardiovascular disease, chronic lung disease and chronic liver disease (Table 1). The median duration of symptoms prior to admission was 6.5 days (IQR = 2.0–9.3 days). The most common symptoms were fever and dyspnea reported in 72 (65.5%) and 67 patients (60.9%), respectively. The frequency of confusion or unresponsiveness at presentation was significantly higher in the dead group compared to the alive group (13 of 47 [27.7%] patients vs 4 of 63 [6.3%] patients, respectively, *P* *=* .002). Diarrhea was more frequent in the alive group than in the dead group (14 of 63 [22.2%] patients vs 3 of 47 [6.4%] patients, respectively, *P* *=* .023). The 3 patients with concurrent infections (*Pneumococcus pneumoniae, Mycoplasma pneumoniae, Pseudomonas aeruginosa*) all died in hospital (*P* *=* .042). The median APACHE II score on admission was 14 (IQR = 10–18), median SOFA score was 5 (IQR = 3–8), median National Early Warning Score (NEWS) was 8 (IQR = 5–10), and median CURB-65 score was 2 (IQR = 1–3). The APACHE II, SOFA, and CURB-65 scores were significantly higher in the dead group compared to the alive group (*P* *<* .001*, P* *=* .012 and *P* *=* .003, respectively). In total, 48 patients (43.6%) underwent chest computed tomography (CT), which primarily showed bilateral opacities, with ground glass opacity observed in 28 patients (58.3%).

**Table 1:** Baseline characteristics of critically ill patients with COVID-19.

	All patients(N = 110)	Survivor,(N = 63)	Mortality,(N = 47)	*P* value
Characteristics				
Age, years	71 (63–78)	69 (62–75)	72 (65–79)	.052
≥65 years	76 (69.1)	40 (63.5)	36 (76.6)	.141
<65 years	34 (30.9)	23 (36.5)	11 (23.4)	
Sex				
Male	67 (60.9)	39 (61.9)	28 (59.6)	.804
Female	43 (39.1)	24 (38.1)	19 (40.4)	
BMI	25 (22–27)	25 (22–27)	24 (21–27)	.809
Smoking status				
Never smoker	77 (70)	46 (73)	31 (66)	.274
Former smoker	28 (25)	13 (20)	15 (31)	
Current smoker	5 (5)	4 (7)	1 (3)	
Comorbidities				
Hypertension	55 (50.0)	31 (49.2)	24 (51.1)	.847
Diabetes	40 (36.4)	20 (31.7)	20 (42.6)	.244
Chronic kidney disease	11 (10)	3 (4.8)	8 (17.0)	.034
Dementia	11 (10)	6 (9.5)	5 (10.6)	.847
Cerebrovascular disease	10 (9.1)	6 (9.5)	4 (8.5)	.855
Malignancy	10 (9.1)	4 (6.3)	6 (12.8)	.247
Cardiovascular disease	9 (8.2)	4 (6.3)	5 (10.6)	.417
Chronic lung disease	9 (8.2)	4 (6.3)	5 (10.6)	.417
Chronic liver disease	5 (4.5)	3 (4.8)	2 (4.3)	.900
Mean duration of symptoms before admission, days	6.5 (2.0–9.3)	7.0 (2.5–10.0)	6.0 (2.5–8.0)	.693
Initial common symptoms				
Fever	72 (65.5)	44 (69.8)	28 (59.6)	.263
Dyspnea	67 (60.9)	36 (57.1)	31 (66.0)	.349
Cough	53 (48.2)	32 (50.8)	21 (44.7)	.526
Fatigue or myalgia	38 (34.5)	23 (36.5)	15 (31.9)	.616
Confusion or Unresponsive	17 (15.5)	4 (6.3)	13 (27.7)	.002
Diarrhea	17 (15.5)	14 (22.2)	3 (6.4)	.023
Vital sign on admission				
Body temperature, °C	37.1 (36.6–38.0)	37.2 (36.6–38.1)	37.0 (36.7–38.0)	.885
Heart rate, beats/min	86 (75–100)	85 (74–96)	88 (78–102)	.443
Respiration rate, beats/min	22 (20–26)	22 (20–27)	22 (20–26)	.191
Systolic BP, mmHg	128 (111–147)	130 (112–144)	128 (112–151)	.875
Diastolic BP, mmHg	78 (67–89)	79 (69–87)	76 (66–90)	.656
Mean arterial BP, mmHg	93 (83–107)	93 (84–105)	93 (80–107)	.932
Other respiratory pathogen infections				
Bacteria	3 (2.7)	0	3 (6.4)	.042
Other viruses	0	0	0	
Scoring system				
APACHE II	14 (10–18)	12 (8–16)	17 (12–21)	<.001
SOFA	5 (3–8)	4 (2–7)	6 (4–10)	.012
NEWS	8 (5–10)	7 (5–9)	8 (5–12)	.269
CURB-65	2 (1–3)	2 (1–2)	2 (1–3)	.003
Radiologic findings				
CT, N (%)	48 (43.6)	29 (46.0)	19 (40.4)	.558
Bilateral infiltration	45 (93.8)	26 (89.7)	19 (100)	.148
Unilateral infiltration	3 (6.3)	3 (10.3)	0	
Ground glass opacity	28 (58.3)	14 (48.3)	14 (73.7)	.218
Mixed pattern	16 (33.3)	12 (41.4)	4 (21.1)	
Consolidation	4 (10.3)	3 (5.3)	1 (8.3)	

Laboratory findings on admission are summarized in Table 2. Of the 110 patients, median leukocyte count was 7070/L (IQR = 5083–10430/L) and median lymphocyte count was 10.7% (IQR = 6.7–19.3%). The median C-reactive protein level was 10.4 mg/dl (IQR = 5.9–16.3 mg/dl; normal range <0.5 mg/dl). The median LDH and NT-proBNP levels were 526 U/L (IQR = 412–824 U/L; normal range <250 U/L) and 522 pg/ml (IQR = 236–1044 pg/ml; normal range = 5–113.2 pg/ml), respectively. The median procalcitonin at 0.19 mmol/L (IQR = 0.09–0.43 mmol/L; normal range <0.5 mml/L) and median lactate at 1.6 mmol/L (IQR = 1.3–2.2 mmol/L; normal range = 0.7–2.1 mmol/L) were within the normal ranges. Blood urea nitrogen (BUN), creatinine, glucose and creatine kinase-MB (CK-MB) values were significantly higher in the dead group compared to the alive group (*P* *=* .004*, P* *=* .001*, P* *=* .044 and *P* *=* .019, respectively). In initial blood gas analysis, median PaO_2_/FiO_2_ ratio (PF ratio) was 137 (IQR = 81–203), and bicarbonate values were significantly lower in the dead group than in the alive group (*P* *=* .003).

**Table 2:** Laboratory findings of patients on admission.

	All patients(N = 110)	Survivor,(N = 63)	Mortality,(N = 47)	*P* value
White blood cell, /L	7070 (5083–10430)	7020 (5320–9535)	7450 (4955–11555)	.679
Neutrophil, %	82.3 (73.8–88.7)	80.7 (74.5–87.7)	84.0 (73.1–89.9)	.288
Lymphocyte, %	10.7 (6.7–19.3)	11.5 (7.0–17.6)	10.1 (5.5–19.9)	.562
Monocyte, %	4.6 (2.9–6.4)	4.6 (3.3–6.4)	4.25 (2.6–6.2)	.389
Hemoglobin, g/dl	12.7 (10.9–14.0)	13.0 (11.5–14.3)	12.2 (10.4–13.9)	.099
Hematocrit, %	37.2 (31.9–40.8)	38.2 (33.6–41.3)	36.3 (30.5–40.1)	.118
Platelet count, 10^3^/L	178 (137–249)	195 (147–251)	166 (125–208)	.150
C-reactive protein, mg/dl	10.4 (5.9–16.3)	10.4 (5.8–14.1)	10.5 (6.4–16.8)	.641
Procalcitonin, mmol/L	0.19 (0.09–0.43)	0.19 (0.09–0.35)	0.18 (0.10–0.76)	.379
Lactate, mmol/L	1.6 (1.3–2.2)	1.6 (1.2–2.2)	1.5 (1.3–2.2)	.618
Albumin, g/dl	3.3 (3.0–3.5)	3.3 (3.0–3.5)	3.2 (2.9–3.5)	.298
Total bilirubin, mg/dl	0.71 (0.42–1.10)	0.67 (0.47–1.16)	0.81 (0.41–1.03)	.937
AST, U/L	50 (32–74)	49 (35–72)	50 (32–73)	.887
ALT, U/L	26 (16–40)	26 (15–43)	27 (16–39)	.681
LDH, U/L	526 (412–824)	553 (417–847)	507 (406–784)	.680
Prothrombin time, INR	1.11 (1.02–1.23)	1.11 (1.02–1.16)	1.11 (1.02–1.30)	.496
BUN, mg/dl	18.1 (13.0–26.2)	16.0 (12.3–22.7)	22.2 (14.7–34.8)	.004
Creatinine, mg/dl	0.97 (0.70–1.32)	0.86 (0.69–1.11)	1.21 (0.78–1.98)	.001
Sodium, mmol/L	136 (133–139)	137 (134–139)	134 (133–139)	.242
Potassium, mmol/L	4.0 (3.5–4.6)	3.9 (3.5–4.4)	4.2 (3.6–4.7)	.163
Glucose, mg/dl	149 (117–192)	141 (111–170)	161 (121–230)	.044
>176	30 (27.3)	11 (17.5)	19 (40.4)	.007
NT-proBNP, pg/ml	522 (236–1044)	416 (228–872)	631 (329–1997)	.141
D-dimer, ug/ml	1.75 (0.94–3.9)	1.47 (0.91–3.07)	2.09 (1.19–5.40)	.332
Troponin I or T, ng/ml	0.02 (0.01–0.10)	0.02 (0.01–0.07)	0.04 (0.02–0.15)	.108
CK-MB, U/L	1.8 (1.0–4.6)	1.7 (1.0–3.0)	2.7 (1.4–6.3)	.019
ABGA				
pH	7.43 (7.35–7.46)	7.43 (7.39–7.47)	7.38 (7.31–7.46)	.027
PaCO_2_, mm Hg	34.1 (30.1–38.4)	34.1 (31.6–37.3)	34.2 (28.5–41.4)	.789
HCO_3_, mmol/L	22.3 (19.0–25.2)	22.9 (21.2–25.6)	20.1 (17.4–23.7)	.003
PF ratio	137 (81–203)	131 (80–206)	142 (83–197)	.978
≤150	63 (57.3)	36 (57.1)	27 (57.4)	.948

### 3.2 Complications and treatment-related outcome

**Figure 1:**
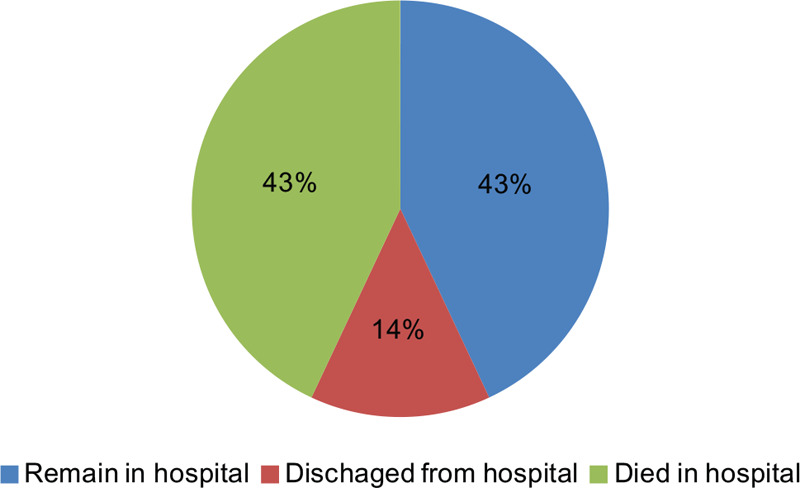
Clinical outcome of critically ill patients with COVID-19.

The most common SARS-CoV-2 infection related complication was ARDS (95 of 110 [86.4%] patients), followed by acute cardiac injury (26 of 76 [34.2%] patients), septic shock (25 of 110 [22.7%] patients) and AKI (21 of 110 [19.1%] patients) (Table 3). The number of patients with ARDS and AKI was significantly higher in the dead group, as compared to the alive group (*P* *=* .002 and *P* *=* .048, respectively). Of the 79 patients (71.8%) who received invasive mechanical ventilation, 46 patients (58.2%) received neuromuscular blockade injection and 19 (24.1%) received ECMO treatment. The number of patients with neuromuscular blockade injection was significantly higher in the dead group compared to the alive group (*P* *=* .021). In total, 75 patients (68.2%) received vasopressors, 21 patients (19%) received renal replacement therapy, and both treatments were significantly more frequent in the dead group than the alive group (*P* *=* .040 and *P* *=* .014, respectively). All patients received antibiotic injection, with 99 patients (90%) receiving hydroxychloroquine, 96 patients (87.3%) receiving lopinavir-ritonavir antiviral medication, 88 patients (80%) receiving glucocorticoid therapy and 14 patients (12.7%) receiving other antiviral agents, such as darunavir-cobicistat and emtricitabine-tenofovir. Thirteen patients received darunavir-cobicistat and 1 patient received emtricitabine-tenofovir. The number of patients who received other antiviral agents was significantly higher in the alive group compared the dead group (*P* *=* .021). At the end of the study period, 48 patients (43.6%) were still hospitalized, 47 patients (42.7%) died in the hospital, and 15 patients (13.6%) were discharged home (Fig. 1). Of the 48 hospitalized patients, 25 patients (52.1%) were in the ICU and 12 (25%) were receiving MV.

**Table 3:** Complications and treatment of critically ill patients with COVID-19.

	All patients(N = 110)	Survivor(N = 63)	Mortality(N = 47)	*P* value
Complications				
ARDS	95 (86.4)	49 (77.8)	46 (97.9)	.002
Septic shock	25 (22.7)	11 (17.5)	14 (29.8)	.127
Acute cardiac injury	26/76 (34.2)	12/45 (26.7)	14/31 (45.2)	.095
Acute kidney injury	21 (19.1)	8 (12.7)	13 (27.7)	.048
ICU therapies				
High-flow nasal cannula	58 (52.7)	34 (54.0)	24 (51.1)	.763
CPAP or NIV	0	0	0	
Invasive MV	79 (71.8)	43 (68.3)	36 (76.6)	.336
Mode Volume control	40/79 (50.6)	26/43 (60.5)	14/36 (38.9)	.056
Pressure control	39/79 (49.4)	17/43 (39.5)	22/36 (61.1)	
PEEP, cmH2O	12 (10–14)	12 (10–12)	12 (10–14)	.544
FIO2, %	95 (61–100)	93 (60–100)	95 (68–100)	.912
Plateau pressure, cmH2O	27 (22–29)	27 (22–29)	27 (23–29)	.995
Prone position	9/79 (11.4)	3/43 (7.0)	6/36 (16.7)	.177
Recruitment maneuver	3/79 (3.8)	2/43 (4.7)	1/36 (2.8)	.664
Neuromuscular blockade	46/79 (58.2)	20/43 (46.5)	26/36 (72.2)	.021
Neuromuscular blockade used > 48 hours	35/79 (44.3)	16/43 (37.2)	19/36 (52.8)	.165
ECMO	19/79 (24.1)	11/43 (25.6)	8/36 (22.2)	.728
Vasopressors	75 (68.2)	38 (60)	37 (78)	.040
Renal replacement therapy	21 (19)	7 (1)	14 (29)	.014
Tracheostomy	28/79 (35.4)	21/43 (48.8)	7/36 (19.4)	.007
Medical treatments				
Antibiotics	110 (100)	63 (100)	47 (100)	
Hydroxychloroquine	99 (90)	58 (92.1)	41 (87.2)	.404
Lopinavir-ritonavir	96 (87.3)	53 (84.1)	43 (91.5)	.252
Other antiviral agents	14 (12.7)	12 (19.0)	2 (4.3)	.021
Glucocorticoid	88 (80)	49 (77.8)	39 (83.0)	.500
Duration of usage, days	12 (6–18)	12 (8–19)	9 (5–16)	.062
Total dose, mg	480 (255–643)	483 (362–613)	455 (178–643)	.292
Medical event				
VAP	13/79 (16.5)	9/43 (20.9)	4/36 (11.1)	.241
CRBSI	8 (7.3)	7 (11.1)	1 (2.1)	.135
DIC/Bleeding	14 (12.7)	8 (12.7)	6 (12.8)	.992
Time from admission to death, discharge or final follow-up^+^, days	28.5 (15.8–42.0)	37.0 (26.0–47.5)	16.0 (6.0–25.5)	<.001

### 3.3 Risk factors analysis

In the univariable analysis, the odds ratio of in-hospital death was higher in patients with diarrhea. APACHE II score, BUN, glucose levels, as well as the use of neuromuscular blockade and other antiviral agents were also associated with death (Table 4). In the multivariable logistic regression model using enter method, the odds ratio of in-hospital death was higher with APACHE II score (OR = 1.126; 95% CI = 1.014–1.252; *P* *=* .027).

**Table 4:** Univariate and multivariate analysis of risk factors associated with in-hospital mortality in critically ill patients with COVID-19.

	Univariable OR(95% CI)	*P* value	Multivariable^∗^ OR(95% CI)	*P* value
Clinical characteristics and laboratory findings				
Diarrhea	0.239 (0.064–0.886)	.032		
APACHE II score	1.129 (1.056–1.207)	<.001	1.126 (1.014–1.252)	.027
BUN, mg/dL				
≤17.6	1 (ref)			
>17.6	3.722 (1.652–8.387)	.002		
Glucose, mg/dl				
≤176	1 (ref)			
>176	3.208 (1.340–7.681)	.009		
PF ratio				
>150	1 (ref)			
≤150	1.026 (0.473–2.225)	.948		
Treatment (vs not implemented)				
Neuromuscular blockade	2.902 (1.324–6.362)	.008		
Antiviral agents other than Lopinavir-ritonavir	0.189 (0.040–0.890)	.035		

## 4 Discussion

The median age of critically ill patients with COVID-19 was 71 years old. During the study period, 47 patients (42.7%) died in the hospital. The most common SARS-CoV-2 infection related complication was ARDS (86.4%) and approximately 72% of all patients required mechanical ventilation. In the multivariable logistic regression model, higher APACHE II score was an independent predictor of in-hospital death.

Previous studies of critically ill patients with COVID-19 have reported varying mortality rates, ranging from 26% to 67%.^[3–5]^ The differences in mortality rate appear to be influenced by social factors such as the regional COVID-19 epidemic situation and the availability of medical personnel and resources. In these studies, the long-term outcome is likely to be worse than reported because many patients were still hospitalized at the time of data analysis. Similarly in our study, more than half of the hospitalized at the end of study period were in ICU and more than a quarter were on mechanical ventilation. The actual in-hospital mortality may be higher than what we investigated. APACHE II score is a well-known and widely used international severity scoring system, with good discriminatory value across a range of disease processes.^[10]^ In this study, significantly higher APACHE II scores were observed in ICU non-survivors compared to those who survived, which aligns with the results of previous studies of critically ill patients with COVID-19.^[5]^ Moreover, studies that analyzed ICU patients with Middle East respiratory syndrome (MERS), which is also caused by coronaviruses, found that the APACHE II scores of non-survivors were higher.^[11–13]^ In addition to APACHE II, this study found that SOFA and CURB-65 scores were significantly higher in non-survivors, whereas NEWS did not differ between groups. This suggests that evaluating the severity of ICU patients based on only vital signs and consciousness, components of NEWS, may be inappropriate. These findings suggest that, as a simple scoring system, CURB-65 may be more effective in identifying critically ill COVID-19 patients with a poor prognosis than NEWS.

Based on previous studies that demonstrated the antiviral effects of lopinavir-ritonavir and chloroquine on SARS-CoV or SARS-CoV-2 in vitro,^[14,15]^ many patients with COVID-19 have received these drugs in clinical settings. However, there have been no studies to prove the effect of these drugs in patients with COVID-19.^[16]^ A recent randomized controlled trial evaluating the efficacy of lopinavir-ritonavir for SARS-CoV-2 infection found no benefit for clinical improvement.^[17]^ In our study, approximately 90% of patients received lopinavir-ritonavir or hydroxychloroquine; however, we were unable to compare the use of these drugs between groups. Anti-viral agents other than lopinavir-ritonavir, such as darunavir-cobicistat, showed significant differences between groups in univariate analysis. In this study, only 13 patients received darunavir-cobicistat treatment; thus, further research is warranted. Despite the limited evidence, corticosteroids are recommended in certain situations, such as refractory shock or ARDS.^[18]^ In the present study, corticosteroid treatment was not associated with the clinical outcome. To date, there are conflicting findings on whether the administration of corticosteroid is associated with faster symptom improvement or reduced risk of death ^[19,20]^; thus, further research on corticosteroid treatment in patients with COVID-19 is required.

This study has several limitations. First, selection bias was unavoidable due to the retrospective nature of the study. Patients transferred to other cities and those deemed “Dead on Arrival” were not included. Second, some specific information from laboratory or radiologic findings was missing. Therefore, the impact of these variables on the prognosis may be underestimated. Third, we adjusted for many potential confounders, but some unmeasured or unknown variables may have influenced these results. Lastly, due to the relatively short follow-up period and the lack of clinical data following discharge, long-term outcomes could not be assessed. Despite these limitations, our study analyzed the data of a relatively large number of patients from the ICUs of 7 hospitals, and these findings provide insight into the clinical features, risk factors and management of critically ill patients with COVID-19.

In conclusion, in this retrospective cohort study involving critically ill patients with COVID-19, the in-hospital mortality rate was found to be approximately 40%. Higher APACHE II score at admission was an independent risk factor for death in these patients.

## Author contributions

**Conceptualization:** Kyeong-Cheol Shin, Eun Young Choi.

**Data curation:** Eun Jin Kim, Yong Hoon Lee, Jae Seok Park, Jaehee Lee, Shin Yup Lee, Yeonjae Kim, Yong Shik Kwon, Jong Geol Jang.

**Formal analysis:** Yong Hoon Lee.

**Funding acquisition:** Eun Young Choi.

**Methodology:** Kyung Chan Kim.

**Writing – original draft:** Eun Jin Kim, Yong Hoon Lee, Jae Seok Park.

**Writing – review & editing:** Eun Young Choi.

## References

[1] Ferreyro BL Angriman F Munshi L. Noninvasive oxygenation strategies in adult patients with acute respiratory failure: a protocol for a systematic review and network meta-analysis. Syst Rev 2020;9:95. 32336293 10.1186/s13643-020-01363-0 PMC7184712

[2] Semler MW Bernard GR Aaron SD. Identifying clinical research priorities in adult pulmonary and critical care: NHLBI working group report. Am J Respir Crit Care Med 2020;202:511–23. 32150460 10.1164/rccm.201908-1595WS PMC7427373

[3] Grasselli G Zangrillo A Zanella A. Baseline characteristics and outcomes of 1591 patients infected with SARS-CoV-2 admitted to ICUs of the Lombardy Region, Italy. JAMA 2020;323:1574–81. 32250385 10.1001/jama.2020.5394 PMC7136855

[4] Arentz M Yim E Klaff L. Characteristics and outcomes of 21 critically ill patients with COVID-19 in Washington State. JAMA 2020;323:1612–4. 32191259 10.1001/jama.2020.4326 PMC7082763

[5] Yang X Yu Y Xu J. Clinical course and outcomes of critically ill patients with SARS-CoV-2 pneumonia in Wuhan, China: a single-centered, retrospective, observational study. Lancet Respir Med 2020;8:475–81. 32105632 10.1016/S2213-2600(20)30079-5 PMC7102538

[6] Ferguson ND Fan E Camporota L. The Berlin definition of ARDS: an expanded rationale, justification, and supplementary material. Intensive Care Med 2012;38:1573–82. 22926653 10.1007/s00134-012-2682-1

[7] Singer M Deutschman CS Seymour CW. The third international consensus definitions for sepsis and septic shock (Sepsis-3). JAMA 2016;315:801–10. 26903338 10.1001/jama.2016.0287 PMC4968574

[8] Mehta RL Kellum JA Shah SV. Acute kidney injury network: report of an initiative to improve outcomes in acute kidney injury. Crit Care 2007;11:R31. 17331245 10.1186/cc5713 PMC2206446

[9] Bellani G Pham T Laffey JG. Missed or delayed diagnosis of ARDS: a common and serious problem. Intensive Care Med 2020;46 (6):1180–3. 32328723 10.1007/s00134-020-06035-0 PMC7176813

[10] Bouch DC Thompson JP. Severity scoring systems in the critically ill. Continuing education in anaesthesia, critical care & pain 2008;8:181–5.

[11] Halim AA Alsayed B Embarak S Yaseen T Dabbous S. Clinical characteristics and outcome of ICU admitted MERS corona virus infected patients. Egypt J Chest Dis Tuberc 2016;65:81–7. 32288128 10.1016/j.ejcdt.2015.11.011 PMC7132710

[12] Almekhlafi GA Albarrak MM Mandourah Y. Presentation and outcome of Middle East respiratory syndrome in Saudi intensive care unit patients. Crit Care 2016;20:123. 27153800 10.1186/s13054-016-1303-8 PMC4859954

[13] Cheng Y Luo R Wang K. Kidney disease is associated with in-hospital death of patients with COVID-19. Kidney Int 2020;97:829–38. 32247631 10.1016/j.kint.2020.03.005 PMC7110296

[14] Wang M Cao R Zhang L. Remdesivir and chloroquine effectively inhibit the recently emerged novel coronavirus (2019-nCoV) in vitro. Cell Res 2020;30:269–71. 32020029 10.1038/s41422-020-0282-0 PMC7054408

[15] Chu C Cheng V Hung I. Role of lopinavir/ritonavir in the treatment of SARS: initial virological and clinical findings. Thorax 2004;59:252–6. 14985565 10.1136/thorax.2003.012658 PMC1746980

[16] Organization WH. Clinical management of severe acute respiratory infection (SARI) when COVID-19 disease is suspected: interim guidance, 13 March 2020. World Health Organization. https://apps.who.int/iris/handle/10665/331446.

[17] Cao B Wang Y Wen D. A trial of lopinavir–ritonavir in adults hospitalized with severe Covid-19. N Engl J Med 2020;382:1787–99. 32187464 10.1056/NEJMoa2001282 PMC7121492

[18] Alhazzani W Møller MH Arabi YM. Surviving sepsis campaign: guidelines on the management of critically ill adults with Coronavirus Disease 2019 (COVID-19). Intensive Care Med 2020;46:854–87. 32222812 10.1007/s00134-020-06022-5 PMC7101866

[19] Wu C Chen X Cai Y. Risk factors associated with acute respiratory distress syndrome and death in patients with coronavirus disease 2019 pneumonia in Wuhan, China. JAMA Intern Med 2020;180:934–43. 32167524 10.1001/jamainternmed.2020.0994 PMC7070509

[20] Wang Y Jiang W He Q. Early, low-dose and short-term application of corticosteroid treatment in patients with severe COVID-19 pneumonia: single-center experience from Wuhan, China. medRxiv 2020;DOI: 10.1101/2020.03.06.20032342.

